# Optimal promising zone designs

**DOI:** 10.1002/bimj.201700308

**Published:** 2018-11-08

**Authors:** Samuel T. Hsiao, Lingyun Liu, Cyrus R. Mehta

**Affiliations:** ^1^ Cytel Corportation Cambridge Massachusetts USA; ^2^ Harvard T.H. Chan School of Public Health Boston Massachusetts USA

**Keywords:** adaptive design, gold standard sample size reassessment rule, group sequential design, optimal adaptive design, power comparisons of adaptive versus nonadaptive, promising zone design, sample size reassessment, trial optimization

## Abstract

Clinical trials with adaptive sample size reassessment based on an unblinded analysis of interim results are perhaps the most popular class of adaptive designs (see Elsäßer et al., 2007). Such trials are typically designed by prespecifying a zone for the interim test statistic, termed the promising zone, along with a decision rule for increasing the sample size within that zone. Mehta and Pocock (2011) provided some examples of promising zone designs and discussed several procedures for controlling their type‐1 error. They did not, however, address how to choose the promising zone or the corresponding sample size reassessment rule, and proposed instead that the operating characteristics of alternative promising zone designs could be compared by simulation. Jennison and Turnbull (2015) developed an approach based on maximizing expected utility whereby one could evaluate alternative promising zone designs relative to a gold‐standard optimal design. In this paper, we show how, by eliciting a few preferences from the trial sponsor, one can construct promising zone designs that are both intuitive and achieve the Jennison and Turnbull (2015) gold‐standard for optimality.

## 1 INTRODUCTION

At the design stage of a clinical trial one determines the required sample size by specifying a treatment effect that is both clinically meaningful and realistic, and ensures that the sample size is large enough to detect the treatment effect with high power. Sometimes, however, there is uncertainty about the magnitude of the treatment effect and a conservative estimate is used, resulting in a sample size that is too large to justify an up‐front commitment to the usual fixed‐sample design. Two alternative approaches are available to resolve this difficulty—group sequential and adaptive. The classical group sequential design starts out with a large sample size, whereby the study is adequately powered to detect small but clinically meaningful treatment effects. The trial is, however, monitored at administratively convenient time points with the possibility of early termination if there is overwhelming evidence of efficacy or futility. The type‐1 error is controlled by the α‐spending methodology of Lan and DeMets (1983). In contrast the adaptive sample size reassessment design starts out with a smaller sample size whereby it is adequately powered to detect a realistic treatment effect that is somewhat larger than the smallest clinically meaningful improvement. There is provision, however, to increase the sample size and thus repower the study at an interim analysis time point, should the observed data make it desirable to do so. In this paper, we consider sample size reassessment methods that utilize unblinded estimates of the actual treatment effect rather than relying solely on blinded estimates of nuisance parameters like interpatient variability (see Proschan, 2009 for further clarity). Several approaches (e.g., Chen, DeMets & Lan, 2006; Cui, Hung, & Wang, 1999; Lehmacher & Wassmer, 1999; Müller & Schäfer 2001; Mehta & Pocock, 2011) have been proposed for controlling the type‐1 error in this setting. One can also design trials that include both group sequential early stopping and adaptive sample size reassessment. The Champion Phoenix trial discussed in Bhatt and Mehta (2016) is an example of such a design.

The relative merits of group sequential versus adaptive sample size re‐assessment designs have been widely discussed (e.g., Emerson, Levin, & Emerson, 2011; Glimm, 2012; Mehta & Liu, 2016; Liu, Hsiao & Mehta, 2018) and will not be repeated here. Instead we will focus on adaptive sample size reassessment designs. Such designs are characterized by a range of values for the interim test statistic (termed the promising zone) within which the sample size may be increased in accordance with a decision rule for determining the magnitude of the increase. The operating characteristics of these designs (hereafter referred to as promising zone designs) depend entirely on the choice of promising zone and the corresponding decision rule. Mehta and Pocock (2011) provided examples of promising zone designs in the neurology and cardiology therapeutic areas. Jennison and Turnbull (2015) took up the neurology example to illustrate several alternative design options including a fixed sample design, a group sequential design with delayed effects and a promising zone design in which the zone and decision rule were determined by optimizing an objective function reflecting the trade‐off between increasing conditional power and paying for it by increasing sample size. The latter design, being optimal, can serve as a useful benchmark against which to compare the operating characteristics of the other candidates.

In this paper, we present a new promising zone design that is easy to implement, and easy for all stake‐holders to understand and accept. It is based on the intuitively plausible notion that any additional investment of sample size at an interim analysis should be contingent on a minimal acceptable return on the investment, expressed in terms of guaranteed conditional power. We shall see that this requirement imposes a constraint on the promising zone, and hence we shall hereafter refer to this design as the “constrained promising zone design” or CPZ design. We shall benchmark the CPZ design against the optimal unconstrained Jennison and Turnbull (2015) design (the JT design) and against a constrained version of the Jennison and Turnbull (2015) design (the CJT design). While the minimal acceptable rate of return might differ, depending on the type of trial and sponsor organization, it is seen that if the same constraint is imposed on both the CPZ and CJT designs, then they have almost identical operating characteristics.

In Section 2, we introduce a clinical trial in advanced pancreatic cancer that will serve as the motivating example for illustrating all the design concepts in this paper. In Section 3, we introduce the new promising zone design in which the promising zone is constrained by the requirement of a minimal return on investment. In Section 4, we describe the optimal Jennison and Turnbull (2015) design. In Section 5, we compare the operating characteristics of the two designs with and without the added constraint of a minimal required return on investment. We conclude in Section 6 with an analysis of the trade‐off between conditional and unconditional power, followed by a more philosophical discussion of whether sponsors, by imposing minimal requirements for conditional power, are being consistent with a sensible utility function.

## 2 MOTIVATING EXAMPLE

In a recent clinical trial of advanced pancreatic cancer (see ClinicalTrials.gov Identifier NCT02715804), patients were randomized between a recombinant human hyaluronidase (the treatment arm) and matching placebo (the control arm). The primary efficacy endpoint was progression‐free survival (PFS). For trials with time to event endpoints such as PFS, the crucial design parameter for power calculations is the hazard ratio (HR). Allowing for the uncertainty in this parameter, it was determined that the trial should be designed to provide adequate power for values of HR in the range 0.67–0.75. At HR = 0.67, the optimistic end of the spectrum, 280 PFS events would be required to achieve about 92% power at a one‐sided α of 0.025. Given a median PFS of 8.5 months for the control arm one can show that with 350 patients enrolled over 28 months the study could be completed in about 40 months with the requisite 280 events. Such a study was well within the resource constraints of the trial sponsor. However these resources would be inadequate at HR = 0.75, the pessimistic end of the spectrum, where the power would deteriorate to 67%. To achieve 90% power at HR = 0.75, 500 PFS events are needed, and hence the study would require a larger sample size and longer follow‐up—for example, one could obtain 500 PFS events by enrolling 600 patients over 36 months and following them for an additional 12 months. Although it might be difficult to make an up‐front commitment to such a large trial, it is entirely practicable to make a milestone‐based investment. In this approach, the financing of the trial would occur in two tranches. The first tranche would suffice to fund the smaller 280 PFS event trial, which would then be adequately powered to detect HR = 0.67. The second tranche would be milestone‐based. At an interim analysis based on the first 140 PFS events, if the conditional power for detecting HR = 0.75 were to fall in a prespecified promising zone, an additional investment would be forthcoming that would permit the trial to remain open until a total of 420 PFS events were obtained. We shall use this example to illustrate our new constrained promising zone design and shall compare its operating characteristics with those of the optimal Jennison and Turnbull (2015) design. Since this is an event‐driven trial, its power depends on number of PFS events rather than number of patients. Accordingly we shall use the term sample size to mean PFS events in all the design specifications below. It will be assumed that the actual patient enrollment suffices to obtain the required number of PFS events in a reasonable time frame. Otherwise patient enrollment will play no further role in the discussions that follow.

## 3 THE CONSTRAINED PROMISING ZONE DESIGN

In the usual set‐up of a two‐arm randomized clinical trial, let δ denote the (unknown) treatment effect, or mean improvement in response on the treatment arm relative to the control arm. We wish to test the null hypothesis $H_{0}:\;\delta =0$ against the one‐sided alternative hypothesis that δ>0. Let $\hat{\delta }$ be its maximum likelihood estimate and *I* be its Fisher information, based on a total of *n* subjects randomized equally to the two arms. Let $Z=\hat{\delta }\sqrt{I}$ be the corresponding Wald statistic. Then, asymptotically, $Z∼N(\delta \sqrt{I},1)$. Although the pancreatic cancer trial has a time to event endpoint it can nevertheless be accommodated in the above framework by fitting a Cox proportional hazards model to the data, with δ as the coefficient of the treatment variable. Let $\hat{\delta }$ be the maximum partial likelihood estimate of δ and let *I* be the corresponding Fisher information. Then by the results of Schoenfeld (1981) it can be shown that I=n/4 asymptotically, where *n* is the number of PFS events, so that $Z=\hat{\delta }\sqrt{n}/2$ is $N(\delta \sqrt{n}/2,1)$. Suppose an interim analysis is performed after *n*_1_ PFS events. Let ${\hat{\delta }}_{1}$, *I*_1,_ and *Z*_1_ be the corresponding interim statistics. Then, by the result of Tsiatis (1981), $Z_{1}\sqrt{I_{1}}$ and $Z\sqrt{I}-Z_{1}\sqrt{I_{1}}$ are independent. These asymptotic results suffice to design the trial as though the accruing patient level data were iid normal with unit variance and a mean difference δ between the control and intervention arms of the trial. Hereafter we will assume as much, will use the term sample size or PFS events interchangeably, and will denote it by the symbol *n*.

At the interim analysis, when *n*_1_ events have arrived and *z*_1_ is the observed value of the Wald statistic, let ${\text{CP}}_{\delta }\left(z_{1},n\right)=P_{\delta }\left(Z\geq z_{\alpha }|z_{1}\right)$ be the conditional power for attaining level‐α statistical significance at the final analysis, where $z_{\alpha }=\Phi^{-1}\left(1-\alpha \right)$. Then, as shown in Gao, Ware, and Mehta (2008),

(1) $${\text{CP}}_{\delta }\left(z_{1},n\right)=\Phi \left\{\frac{\delta \sqrt{n-n_{1}}}{2}-\frac{z_{\alpha }\sqrt{n}-z_{1}\sqrt{n_{1}}}{\sqrt{n-n_{1}}}\right\}\;.$$

If, based on the results of the interim analysis, the sample size is increased from *n* to $n^{*}$, then the final analysis must be adjusted appropriately to preserve the type‐1 error. One may adjust either the final test statistic, as proposed by Cui et al. (1999), or the final critical value, as proposed by Müller and Shäfer (2001). In either case, by the results in Gao et al. (2008), the conditional power formula is altered to

(2) $${\text{CP}}_{\delta }\left(z_{1},n^{*}\right)=\Phi \left\{\frac{\delta \sqrt{n^{*}-n_{1}}}{2}-\frac{z_{\alpha }\sqrt{n}-z_{1}\sqrt{n_{1}}}{\sqrt{n-n_{1}}}\right\}$$

with *n* being replaced by $n^{*}$ only in the numerator of the first term but nowhere else.

Since the true treatment effect δ is unknown, it is usual to either substitute an estimate derived from the interim data or to use a constant having desirable properties. The constant $\delta_{\min}$ – representing the smallest clinically meaningful value of δ in the range of interest – is a reasonable choice for evaluating conditional power. Thereby if the true value of δ were greater than $\delta_{\min}$ the conditional power could only increase, whereas the conditional power at smaller values of δ would be outside the range of interest. For the pancreatic cancer trial, for example, one would choose $\delta_{\min}=-\ln\left(0.75\right)=0.29$.

We may partition the interim analysis results into three zones on the basis of ${\text{CP}}_{\delta_{\min}}\left(z_{1},n\right)$. The range ${\text{CP}}_{\delta_{\min}}\left(z_{1},n\right)<L$ constitutes the unfavorable zone. If the conditional power at the interim look falls in this zone, the sample size will remain *n*. The range $L\leq {\text{CP}}_{\delta_{\min}}\left(z_{1},n\right)\leq U$ constitutes the promising zone. Within this zone the total sample size will be increased from *n* to $n^{*}\left(z_{1}\right)$ in accordance with some prespecified function $n^{*}\left(.\right)$. The range ${\text{CP}}_{\delta_{\min}}\left(z_{1},n\right)>U$ constitutes the favorable zone. Within this zone the sample size will remain *n*. It is convenient to also specify the promising zone in terms of the Wald statistic. To that end let $z_{1}^{\left(L\right)}$ be such that ${\text{CP}}_{\delta_{\min}}\left(z_{1}^{\left(L\right)},n\right)=L$ and let $z_{1}^{\left(U\right)}$ be such that ${\text{CP}}_{\delta_{\min}}\left(z_{1}^{\left(U\right)},n\right)=U$. Then the promising zone is the interval $\left[z_{1}^{\left(L\right)},z_{1}^{\left(U\right)}\right]$. What distinguishes one promising zone design from another is the choice of this interval and the decision rule $n^{*}\left(z_{1}\right)$ for increasing the sample size inside it.

Most sponsors of adaptive trials want to achieve specific milestones at an interim analysis before agreeing to increase the sample size. In this paper, we shall investigate a milestone‐based promising zone design in which the promising zone $\left[z_{1}^{\left(L\right)},z_{1}^{\left(U\right)}\right]$ and the corresponding decision rule $n^{*}\left(z_{1}\right)$ are completely specified by the following constrained optimization problem:

$$\begin{array}{ccc} & & \text{Constrained}\;\text{Promising}\;\text{Zone}\;\text{(CPZ)}\;\text{Design}\\ & & \text{Objective:}\;\text{Maximize}\;\left\{{\text{CP}}_{\delta_{\min}}\left(z_{1},n^{*}\right)\right\}\;\text{by}\;\text{choice}\;\text{of}\;n^{*}\;\text{for}\;\text{all}\;z_{1},\;\text{subject}\;\text{to}\\ & & \text{Constraint}\;\text{1:}\;n\leq n^{*}\leq n_{\max}\\ & & \text{Constraint}\;\text{2:}\;{\text{CP}}_{\delta_{\min}}\left(z_{1},n^{*}\right)\geq {\text{cp}}_{\min}\\ & & \text{Constraint}\;\text{3:}\;{\text{CP}}_{\delta_{\min}}\left(z_{1},n^{*}\right)\leq {\text{cp}}_{\max} \end{array}$$

where $n_{\max}$, cp_min _ , and cp_max _ are specified below and implicitly define the promising zone $\left[z_{1}^{\left(L\right)},z_{1}^{\left(U\right)}\right]$ within which the sample size may be modified.

$n_{\max}$ is the maximum to which the initial sample size *n* may be increased. This constraint implies that there is a limit above which it becomes impractical for an organization to continue investing in the trial.
cp_min _ is the minimum requirement for conditional power inside the promising zone. Unless the conditional power, evaluated at $\delta =\delta_{\min}$, can be boosted to at least cp_min _ by increasing the sample size from *n* to $n_{\max}$, the interim result is considered to be in the unfavorable zone and there is no sample size increase. Thus the value of *z*_1_ at which ${\text{CP}}_{\delta_{\min}}\left(z_{1},n_{\max}\right)={\text{cp}}_{\min}$ determines $z_{1}^{\left(L\right)}$ the start of the promising zone.
cp_max _ is the maximum requirement for conditional power inside the promising zone. It is unnecessary to increase the sample size any more than would be needed for the conditional power, evaluated at $\delta =\delta_{\min}$, to exceed cp_max _. Due to the monotonicity of ${\text{CP}}_{\delta }\left(x,y\right)$ with *x* and *y*, as *z*_1_ increases inside the promising zone, ${\text{CP}}_{\delta_{\min}}\left(z_{1},n_{\max}\right)$ also increases while the sample size remains $n_{\max}$ until at some value, $z_{1}=z_{1}^{\left(C\right)}$ say, ${\text{CP}}_{\delta_{\min}}\left(z_{1}^{\left(C\right)},n_{\max}\right)={\text{cp}}_{\max}$. Thereafter, for values of $z_{1}>z_{1}^{\left(C\right)}$ inside the promising zone, the sample size decreases in accordance with a function $\tilde{n}\left(z_{1}\right)$ defined by ${\text{CP}}_{\delta_{\min}}\left(z_{1},\tilde{n}\left(z_{1}\right)\right)={\text{cp}}_{\max}$, until at $z_{1}=z_{1}^{\left(U\right)}$, $\tilde{n}\left(z_{1}^{\left(U\right)}\right)=n$. For all values of $z_{1}>z_{1}^{\left(U\right)}$ the sample size remains *n*. Thus $z_{1}^{\left(U\right)}$ marks the end of the promising zone.

We shall refer to this design as the “constrained” promising zone design (or CPZ design). It is easy to show that the sample size function for this design is given by

n cpz ∗(z1,δmin,cpmin)=nifz1<z1(L)nmaxifz1(L)≤z1≤z1(C)n∼(z1)ifz1(C)<z1≤z1(U)nifz1>z1(U)

**Figure 1:**
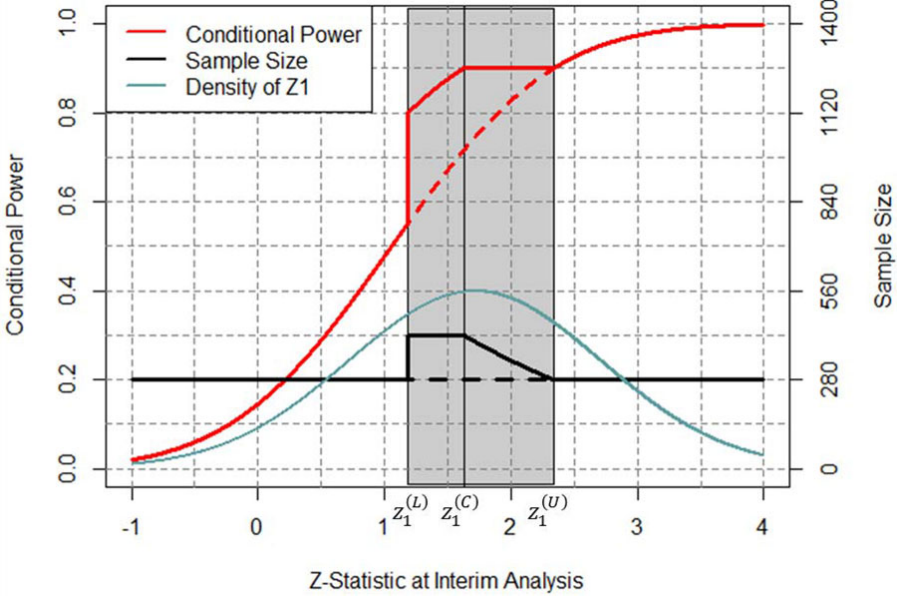
Conditional power and sample size of CPZ design at $\delta_{\min}=0.29$ and ${\text{cp}}_{\min}=0.8$

The dependence of the sample size function on $\delta_{\min}$ and cp_min _ is implicit and arises from the fact that $z_{1}^{\left(L\right)},z_{1}^{\left(C\right)}$ , and $z_{1}^{\left(U\right)}$ all depend on $\delta_{\min}$ and cp_min _. (To avoid cumbersome notation we have not included $n_{\max}$ or cp_max _ as arguments of n cpz ∗.) Figure 1 displays the CPZ design for the pancreatic cancer example with $\delta_{\min}=0.29$, $n_{1}=140$, n=280, $n_{\max}=420$, ${\text{cp}}_{\min}=0.8$, ${\text{cp}}_{\max}=0.9$, and α=0.025. The conditional power graph is displayed in red and the sample size graph is displayed in black. Observe that at $z_{1}=z_{1}^{\left(L\right)}$, the start of the promising zone, the sample size increases from *n* to $n_{\max}$ and the conditional power is boosted to cp_min _. As *z*_1_ increases further the conditional power continues to climb until, at $z_{1}=z_{1}^{\left(C\right)}$, it reaches its peak of cp_max _. Thereafter the conditional power remains constant while the sample size begins to decline until, at $z_{1}=z_{1}^{\left(U\right)}$, the promising zone ends and the sample size is once again equal to *n*. Outside the promising zone the conditional power increases with *z*_1_ in accordance with equation (1). The density of *z*_1_ at δ=0.29 is also displayed in Figure 1.

## 4 OPTIMAL JENNISON AND TURNBULL DESIGNS

The Jennison and Turnbull design (hereafter referred to as the JT design) is also characterized by a promising zone and a corresponding decision rule for increasing the sample size within the promising zone. In this case, however, the promising zone and the corresponding sample size rule are derived implicitly by solving the following optimization problem at any $\delta =\delta_{0}$.

$$\begin{array}{ccc} & & \text{JT}\;\text{Design}\\ & & \text{Objective:}\;\text{Maximize}\;\left\{{\text{CP}}_{\delta_{0}}\left(z_{1},n^{*}\right)-\gamma n^{*}\right\}\;\text{by}\;\text{choice}\;\text{of}\;n^{*}\;\text{for}\;\text{all}\;z_{1},\;\text{subject}\;\text{to}\;\\ & & \text{Constraint:}\;n\leq n^{*}\leq n_{\max},\;\text{where}\;n_{\max}\;\text{is}\;\text{the}\;\text{maximum}\;\text{allowable}\;\text{sample}\;\text{size.} \end{array}$$

The form of the objective function reflects the net gain in conditional power per unit increase in sample size. The constant γ may be interpreted as a tuning parameter or exchange rate between increasing CP and paying for it by increasing $n^{*}$. We discuss its role below and also in Section 5. Here the only explicit constraint is that the sample size may not be decreased, and should not exceed $n_{\max}$. The resulting sample size rule depends on $z_{1},\delta_{0,}$ and γ and is denoted by n jt ∗(z1,δ0,γ). (For notational convenience we have not included $n_{\max}$ as an argument of n jt ∗.) The promising zone interval [L,U] (or equivalently $\left[z_{1}^{\left(L\right)},z_{1}^{\left(U\right)}\right]$) is specified implicitly by the above objective function and sample size constraint. The expected value of the JT objective function at $\delta =\delta_{0}$ is

(3) ∫−∞∞CPδ0z1,n jt ∗(z1,δ0,γ)−γn jt ∗(z1,δ0,γ)fδ0(z1)dz1=Pδ0,γ(RejectH0)−γEδ0,γ(N),

where, both here and elsewhere, the symbol N represents the random sample size whose expectation is being computed. The first term on the right hand side of (3) is the unconditional power of the JT design while the second term is γ times its expected sample size. Since the JT objective function is maximized for every value of *z*_1_, it follows that

(4) $$P_{\delta_{0},\gamma }\left(\text{Reject}H_{0}\right)-\gamma E_{\delta_{0},\gamma }\left(N\right)\;\text{is}\;\text{at}\;\text{its}\;\text{maximum}\;\text{for}\;a\;\text{given}\;\delta_{0}\;\text{and}\;\gamma .$$

Therefore any promising zone design with the same sample size constraint and having the same $E_{\delta_{0},\gamma }\left(N\right)$ must have unconditional power no larger than $P_{\delta_{0},\gamma }\left(\text{Reject}H_{0}\right)$. It follows that if $\delta =\delta_{0}$ then, among all promising zone designs that have the same initial sample size *n*, maximum sample size $n_{\max}$, and expected sample size $E_{\delta_{0},\gamma }\left(N\right)$, the JT design is optimal in terms of unconditional power.

One can consider a modification of the JT design by adding the constraint that the sample size should only be increased if by doing so the conditional power at $\delta =\delta_{\min}$ reaches cp_min _.

$$\begin{array}{ccc} & & \text{Constrained}\;\text{JT}\;\text{(or}\;\text{CJT)}\;\text{Design}\\ & & \text{Objective:}\;\text{Maximize}\;\left\{{\text{CP}}_{\delta_{0}}\left(z_{1},n^{*}\right)-\gamma n^{*}\right\}\;\text{by}\;\text{choice}\;\text{of}\;n^{*}\;\text{for}\;\text{all}\;z_{1},\;\text{subject}\;\text{to}\;\\ & & \text{Constraint}\;\text{1:}\;n\leq n^{*}\leq n_{\max}\\ & & \text{Constraint}\;\text{2:}\;{\text{CP}}_{\delta_{\min}}\left(z_{1},n^{*}\right)\geq {\text{cp}}_{\min}. \end{array}$$

The resulting sample size rule depends on $z_{1},\delta_{0},\gamma ,\delta_{\min,}$ and cp_min _ and is denoted by n cjt ∗(z1,δ0,γ,δmin,cpmin). (Again, for notational convenience, we have not included $n_{\max}$ as an argument of n cjt ∗.) The expected value of the CJT objective function at $\delta =\delta_{0}$ is

∫−∞∞CPδ0(z1,n cjt ∗(z1,δ0,γ,δmin,cpmin))−γn cjt ∗(z1,δ0,γ,δmin,cpmin)fδ0(z1)dz1=Pδ0,γ,δmin,cpmin(RejectH0)−γEδ0,γ,δmin,cpmin(N).

By the same reasoning as was used for the JT design, if $\delta =\delta_{0}$ then among all promising zone designs that have the same $n,n_{\max},\delta_{\min},{\text{cp}}_{\min}$, and expected sample size $E_{\delta_{0},\gamma ,\delta_{\min},{\text{cp}}_{\min}}\left(N\right)$, the CJT design is optimal in terms of unconditional power.

To actually create a JT or CJT design one would have to specify a value for γ by balancing the costs of a higher sample size against the resulting benefits. This could be a challenge for a complex organization with multiple priorities. Therefore, rather than create a stand‐alone JT design, Jennison and Turnbull (2015) used its optimality properties to benchmark other candidate designs relative to the best that could be achieved. To be specific, they used γ as a “tuning parameter” to match $P_{\delta_{0},\gamma }\left(\text{Reject}\;H_{0}\right)$ with the unconditional power of the neurology clinical trial (Mehta & Pocock, 2011) at a specific $\delta =\delta_{0}$ and thereby evaluated the extent of the saving in expected sample size for the JT design. In Section 5, we shall follow a similar approach, but will match the expected sample sizes of the JT and CJT designs with the expected sample size of the CPZ design by appropriate choice of γ. We will then compare the unconditional power of the CPZ design to the corresponding unconditional power of the JT and CJT designs at $\delta =\delta_{0}$ for all δ_0_ in the range of interest.

## 5 BENCHMARKING CPZ AGAINST JT AND CJT DESIGNS

We first compare the operating characteristics of the CPZ design to those of the JT design. To do so in a fair way we shall first equate the expected sample sizes of the two designs for each $\delta =\delta_{0}$ in the range of interest and then compare the two unconditional power curves. The expected sample size of the CPZ design, evaluated at any δ_0_ is

Eδ0,δmin,cpmin(cpz)(N)=∫−∞∞n cpz ∗(z1,δmin,cpmin)fδ0(z1)dz1.

In order to benchmark the unconditional power of the CPZ design relative to the gold‐standard JT design we must equate their expected sample sizes. This is achieved by finding the value of $\gamma^{*}$ such that

(5) ∫−∞∞n jt ∗(z1,δ0,γ∗)fδ0(z1)dz1=Eδ0,δmin,cpmin(cpz)(N),

where, for notational convenience, the dependence of $\gamma^{*}$ on δ_0_ has been suppressed. We can then evaluate the unconditional power of the CPZ and JT designs by integrating over their respective conditional power functions at $\delta =\delta_{0}$. Thus

(6) Pδ0,δmin,cpmin(CPZrejectsH0)=∫−∞∞CPδ0(z1,n cpz ∗(z1,δmin,cpmin))fδ0(z1)dz1

and

(7) Pδ0,γ∗(JTrejectsH0)=∫−∞∞CPδ0(z1,n jt ∗(z1,δ0,γ∗))fδ0(z1)dz1.

**Figure 2:**
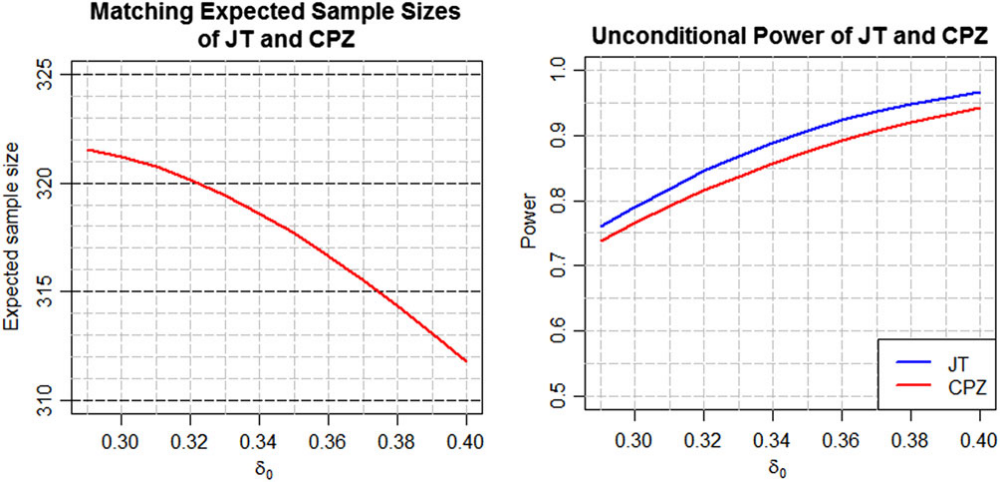
Unconditional power comparison of JT and CPZ designs at the same E(N)

By construction $P_{\delta_{0},\gamma^{*}}\left(\text{JT}\;\text{rejects}\;H_{0}\right)$ must be optimal at $\delta =\delta_{0}$. Thus the extent to which $P_{\delta_{0},\delta_{\min},{\text{cp}}_{\min}}\left(\text{CPZ}\;\text{rejects}\;H_{0}\right)$ falls short can be assessed at every δ_0_ in the range of interest. This is shown in Figure 2 for the pancreatic cancer example for all $\delta_{0}\in \left[0.29,0.4\right]$, $\delta_{\min}=0.29$ and ${\text{cp}}_{\min}=0.8$. The graph on the left displays the expected sample size, which is the same for both designs by construction. The graph on the right shows that the unconditional power of the optimal JT design is about 2–3% higher than that of the CPZ design for all values of δ_0_ in the range of interest. It is important to note that the results in Figure 2 are for comparing **one** CPZ design, in which the promising zone $\left[z_{1}^{\left(L\right)},z_{1}^{\left(U\right)}\right]$ and sample size increase rule n cpz ∗(z1,δmin,cpmin) are fixed by choice of ${\text{cp}}_{\min}=0.9$ at $\delta_{\min}=0.29$, to a **family** of JT designs, one design for each $\delta_{0}\in \left[0.29,0.4\right]$, where every such design is optimized at a different $\gamma^{*}$ that depends on the corresponding δ_0_. This is the only way to ensure that that the expected sample size curves of the two designs will match, albeit by giving JT a small advantage in terms of unconditional power when $\delta_{0}\neq \delta_{\min}$.

**Figure 3:**
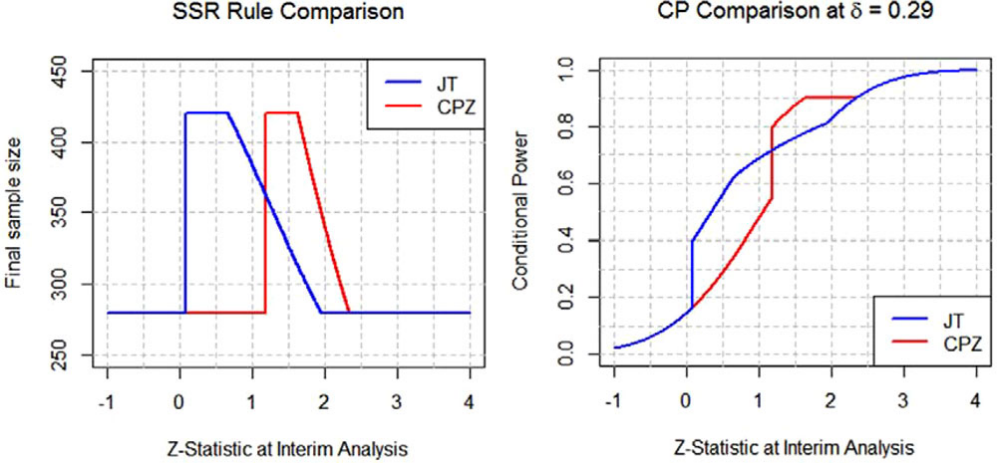
Sample size and conditional power comparison of JT and CPZ at $\delta_{0}=\delta_{\min}=0.29$

Unconditional power, however, is not the only criterion that sponsors of clinical trials consider when deciding whether to increase the sample size. We next examine the operating characteristics of the two designs at $\delta_{0}=\delta_{\min}=0.29$, conditional on the value of *z*_1_ obtained at the interim analysis. The left panel of Figure 3 plots the sample size functions n jt ∗(z1,δmin,γ∗) and n cpz ∗(z1,δmin,cpmin), for the JT and CPZ designs respectively, while the right panel plots the corresponding conditional power functions CPδmin(z1,n jt ∗(z1,δmin,γ∗)) and CPδmin(z1,n cpz ∗(z1,δmin,cpmin)), for $\delta_{\min}=0.29$, ${\text{cp}}_{\min}=0.8$ and *z*_1_ between −1 and 4. The promising zone of the JT design starts earlier than that of the CPZ design and has a wider interval. However, the JT design does not satisfy the one property that is desired by many proponents of adaptive sample size increase—that the sample size should only be increased if by doing so one can attain a conditional power at least equal to cp_min _. It is seen that although the sample size increases from 280 to 420 PFS events at the start of the promising zone, the conditional power under the JT design only jumps from 16 to 39%. This would not be considered an acceptable rate of return for most trial sponsors. Their main purpose for adding resources at the interim analysis time point is to lower the risk of trial failure to an acceptable level. In contrast the conditional power of the CPZ design jumps from 55 to 80% as soon as *z*_1_ enters the promising zone. Thus although the CPZ design falls about 2–3% short of the benchmark in terms of unconditional power it might actually be the preferred option because of its superior conditional profile.

We next compare the operating characteristics of the CPZ design to those of the CJT design. To do this in a fair way we equate the expected sample sizes of the two designs by finding the value of $\gamma^{*}$ at which

**Figure 4:**
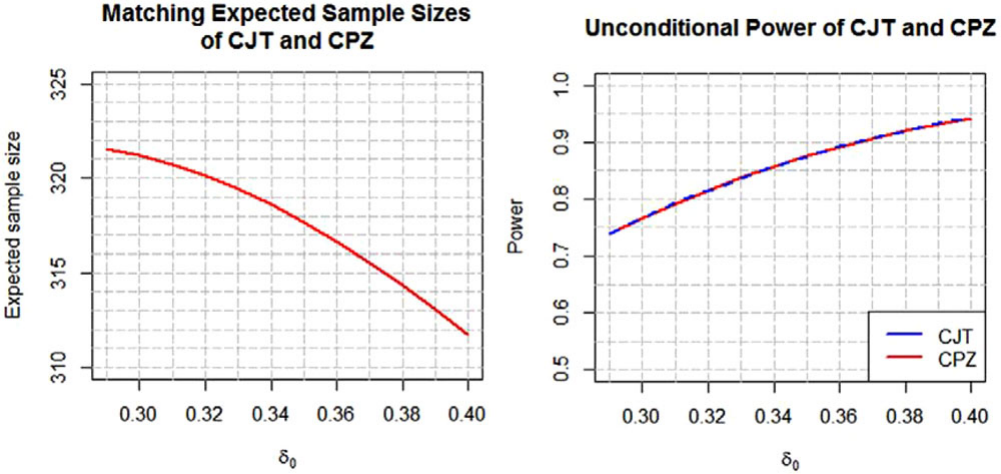
Unconditional power comparison of constrained JT and CPZ at the same E(N)

∫−∞∞n cjt ∗(z1,δ0,γ∗,δmin,cpmin)fδ0(z1)dz1=Eδ0,δmin,cpmin(cpz)(N)

for ${\text{cp}}_{\min}=0.8$ at $\delta_{\min}=0.29$ and every $\delta_{0}\in \left[0.29,0.4\right]$. The resulting plots of the two expected sample size curves are displayed in the left panel of Figure 4. By construction the plots overlap. The right panel of Figure 4 displays the corresponding unconditional power curves for the two designs, evaluate by computing

(8) Pδ0,δmin,cpmin(CPZrejectsH0)=∫−∞∞CPδ0(z1,n cpz ∗(z1,δmin,cpmin))fδ0(z1)dz1

and

(9) Pδ0,γ∗,δmin,cpmin(CJTrejectsH0)=∫−∞∞CPδ0(z1,n cjt ∗(z1,δ0,γ∗,δmin,cpmin))fδ0(z1)dz1

for ${\text{cp}}_{\min}=0.8$, $\delta_{\min}=0.29$ and all $\delta_{0}\in \left[0.29,0.4\right]$. It is seen that the two unconditional power curves match over the entire range of δ_0_ values implying that the CPZ design is optimal with respect to unconditional power in the class of all promising zone designs that have the same required milestone cp_min _ at $\delta =\delta_{\min}$, initial sample size *n*, maximum sample size $n_{\max}$, and expected sample size $E_{\delta_{0},\delta_{\min},{\text{cp}}_{\min}}^{\left(\text{cpz}\right)}\left(N\right)$.

**Figure 5:**
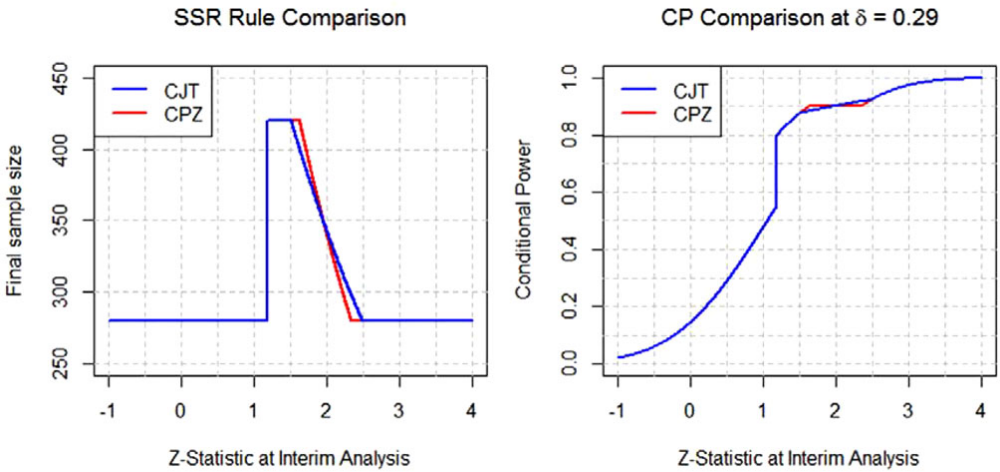
Sample size and conditional power of CJT and CPZ at δ=0.29

We finally examine the operating characteristics of the two designs at $\delta_{0}=\delta_{\min}$, conditional on the value of *z*_1_ obtained at the interim analysis. The left panel of Figure 5 plots the sample size functions n cjt ∗(z1,δmin,γ∗,δmin,cpmin) and n cpz ∗(z1,δmin,cpmin), for the CJT and CPZ designs, respectively, while the right panel plots the corresponding conditional power functions CPδmin(z1,n cjt ∗(z1,δmin,γ∗,δmin,cpmin)) and CPδmin(z1,n cpz ∗(z1,δmin,cpmin)), at $\delta_{\min}=0.29$, ${\text{cp}}_{\min}=0.8$, and *z*_1_ between ‐1 and 4. Both designs have the desirable property of guaranteeing that the conditional power should be at least ${\text{cp}}_{\min}=0.8$ in the promising zone. Also, the sample size and conditional power plots almost overlap; the slight discrepancy is due to the imposition of the ${\text{cp}}_{\max}=0.9$ constraint on the CPZ design but not on the CJT design.

The conditional operating characteristics displayed in Figures 3 and 5 were evaluated at $\delta_{0}=\delta_{\min}=0.29$. We have, however, examined the conditional operating characteristics at other values of $\delta_{0}\in \left[0.29,0.4\right]$ and have obtained qualitatively similar results.

## 6 DISCUSSION

**Figure 6:**
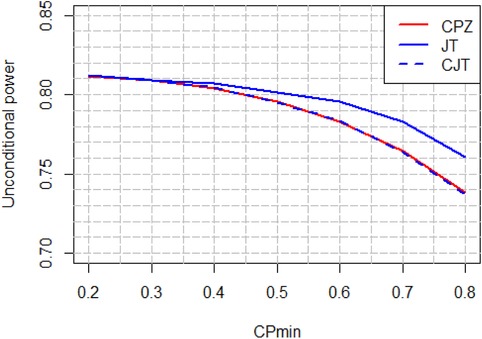
Unconditional power versus cp_min _ at $\delta_{0}=0.29$

The constrained promising zone design for making mid‐course corrections to the sample size in an on‐going trial is intuitive, easy to construct and easy to explain. The design is attractive to sponsors because it guarantees that additional sample size resources will be committed to the trial only if, after an interim analysis, the conditional power exceeds a minimum threshold at the smallest clinically meaningful treatment effect. Moreover we were able to show, with the help of tools developed by Jennison and Turnbull (2015), that among all promising zone designs that share the same constraints for initial sample size, maximum sample size, and minimum required conditional power, the CPZ design is optimal in terms of unconditional power. It is, however, interesting to study how the optimal unconditional power profile changes with the cp_min _ constraint. This is shown in Figure 6 where the unconditional power at $\delta_{0}=\delta_{\min}=0.29$ is plotted against the cp_min _ constraint evaluated at $\delta_{\min}=0.29$, for the CPZ design, the CJT design and the unconstrained JT design, all sharing a common expected sample size at each value of cp_min _ in the range 0.2–0.8. In these plots the CPZ design is first created for a given cp_min _ constraint. Next the CJT design is created at the same cp_min _ constraint with γ selected so as to match the expected sample size of the CPZ design. Finally, the unconstrained JT design is created by disabling the cp_min _ constraint and selecting a different γ so as to match the expected sample size of the other two designs.

Consistent with the results in Section 5 the unconditional power curves for the CPZ and CJT designs overlap completely while the unconditional power curve of the unconstrained JT design dominates over the other two. In all three cases, however, the unconditional power increases with decreasing cp_min _. One could therefore question why a sponsor might prefer a design that had a higher cp_min _ constraint over one with a lower cp_min _ constraint or indeed over the unconstrained JT design, which has the highest unconditional power of all. There is no single correct answer to this question. The perspective of a large pharmaceutical company running numerous clinical trials might differ from that of a small pharmaceutical or biotechnology company with just one product in phase 3 testing. The former can afford to take the long view and opt for designs that maximize unconditional power, notwithstanding their relatively unfavorable conditional operating characteristics, while the latter, with responsibility for one medical compound only, might feel that unconditional power is irrelevant once the study is underway, and might prefer a strategy that gives a larger boost to conditional power if promising interim results are achieved. In this respect, Figure 6 can be used to evaluate the trade‐off between choosing a higher cp_min _ constraint or higher unconditional power when designing a trial with adaptive sample size reassessment. For the pancreatic cancer trial, for example, there is a 4% gain in unconditional power (74 to 78%) if a CPZ design with ${\text{cp}}_{\min}=0.6$ is selected rather than a CPZ design with ${\text{cp}}_{\min}=0.8$. On the other hand the sponsor might not feel comfortable increasing the sample size by 280 to 420 PFS events unless at least 80% conditional power can be achieved by doing so.

The power curve in Figure 6 for the unconstrained JT design displays the best that could be achieved, in terms of unconditional power, if the corresponding cp_min _ constraint were disabled. For example, there is a 2% additional gain in unconditional power (74–76%) relative to a CPZ design with ${\text{cp}}_{\min}=0.8$, and a 1.5% additional gain in unconditional power (78 to 79.5%) relative to a CPZ design with ${\text{cp}}_{\min}=0.6$, if the respective cp_min _ constraints are disabled. It should be noted, however, that in these settings the corresponding minimal conditional power that can be achieved is small and may be unacceptable to the trial sponsor. For example, we can show that for the CPZ design with ${\text{cp}}_{\min}=0.6$, the corresponding unconstrained JT design has a gain in conditional power from 9 to 26% at the cost of a 50% increase in sample size, making this design less compelling to the sponsor at the interim analysis time point notwithstanding its superior unconditional power.

There exists some confusion regarding the use of the term “promising zone design.” The term first appeared in the paper by Mehta and Pocock (2011) where they extended the results of Chen, DeMets, and Lan (2004) and obtained a wider range for the interim test statistic over which the sample size could be increased without having to adjust the conventional Wald statistic at the final analysis. Although not their intent, this wider range of interim values has been interpreted to be the promising zone. We wish to clarify that the term promising zone is not in any way linked to the adjustment needed to perform a valid level‐α test at the final analysis. Rather any region for the interim statistic in which the sample size is increased should be considered a promising zone, and the corresponding design should be termed a promising zone design. Whether or not it is necessary to adjust the final test statistic is a separate matter. In the current work, we do not impose any restriction on the promising zone of the type that was used to extend the results of Chen et al. (2004) by Mehta and Pocock (2011). Therefore if the sample size is increased, the final analysis must utilize the Cui et al. (1999) adjustment or, equivalently, the Müller and Schäfer (2001) adjustment. The equivalence between these two types of adjustments for sample size reassessment was shown by Mehta (2016). These adjustments are incorporated in the formula (2) for conditional power under a sample size increase. This formula is derived from results in Gao et al. (2008).

A referee has asked whether we might comment on the efficiency of our earlier adaptive designs relative to the optimal designs proposed in the current paper. To address this question we revisited the neurology trial that was presented in Mehta and Pocock (2011) and subsequently discussed by Jennison and Turnbull (2015) who termed it the MP design. The primary endpoint of the MP design was the negative symptoms assessment (NSA), a standardized score for measuring symptoms of schizophrenia. The smallest clinically meaningful treatment effect was $\delta_{\min}=1.6$ with σ=7.5. The relevant sample sizes were n=442, and $n_{\max}=884$ with an interim analysis at $n_{1}=208$. The promising zone was specified by conditional power between 0.365 and 0.8, where conditional power was evaluated at the maximum likelihood estimate for δ. The sample size rule called for increasing the sample size so as to achieve a desired CP of 0.8. To assess the efficiency of this design we constructed a CPZ design with matching sample size constraints, and ${\text{cp}}_{\min}=0.8$ at $\delta_{\min}=1.6$, the smallest clinically meaningful treatment effect. The promising zone was obtained by grid search so as to match the expected sample size of the MP design at each value of δ_0_ in the range of interest. Table 1 displays the expected sample size and unconditional power of the MP and CPZ designs for values of δ_0_ between 1.6 and 2.0. The table shows that the MP design suffered about a 2% power loss relative to the optimal CPZ design with ${\text{cp}}_{\min}=0.8$.

**Table 1:** Unconditional power of MP and CPZ designs keeping E(N) the same

δ_0_	MP Pwr	CPZ Pwr	MP E(N)	CPZ E(N)
1.6	65%	67%	499	499
1.7	71%	72%	498	498
1.8	75%	77%	497	496
1.9	79%	81%	494	494
2.0	83%	84%	491	492

A major difference between the CPZ designs and the designs that were presented in Mehta and Pocock (2011) is the manner in which conditional power is evaluated. In Mehta and Pocock (2011) conditional power was evaluated at the estimated value of δ. This has been criticized by Glimm (2012) and also by Emerson et al. (2011) in their respective commentaries on the paper by Mehta and Pocock (2011). Glimm (2012) pointed out that $\hat{\delta }$, the MLE of δ, was used twice; once in the computation of ${\text{CP}}_{\hat{\delta }}\left(z_{1},n\right)$ and again in the computation of *z*_1_. Since $\hat{\delta }$ is a random variable, Glimm was concerned that its double use might lead to undesirable trial modifications and he recommended careful inspection of the operating characteristics of such designs. This concern does not arise for CPZ designs because in their case conditional power is evaluated at $\delta_{\min}$, the smallest clinically meaningful value of δ. Sometimes, however, a trial sponsor might not be able to specify $\delta_{\min}$ due to insufficient information on the new therapy or disease. In that case, the use of $\hat{\delta }$ might be a reasonable approach and a comparison with CPZ designs with different choices for cp_min _ could be used as a sensitivity analysis to determine the robustness of the operating characteristics. We have seen for example from Table 1, that the MP design, in which that target conditional power of 0.8 was evaluated by the MLE, lost only about 2% power relative to a matching CPZ design with the same target conditional power evaluated at the smallest clinically meaningful treatment effect.

This paper has focused exclusively on statistical considerations. Many operational and regulatory issues relating to timeliness of data, preservation of confidentiality, operational bias, and communication with investigators must also be addressed before an adaptive design can be implemented. The guidance documents on adaptive designs by the FDA (2010), FDA (2015), and the EMEA (2007), are excellent sources of information on these issues.

We thank the referees for excellent comments that have greatly strengthened this paper.

## CONFLICT OF INTEREST

The authors have declared no conflict of interest.

## Supporting information

Supporting Information
