# Supplementary figures and images for: Optimal promising zone designs
Source: Biom J. 2018 Nov 8;61(5):1175–86. doi: 10.1002/bimj.201700308 (PMC6767001; doi:10.1002/bimj.201700308)

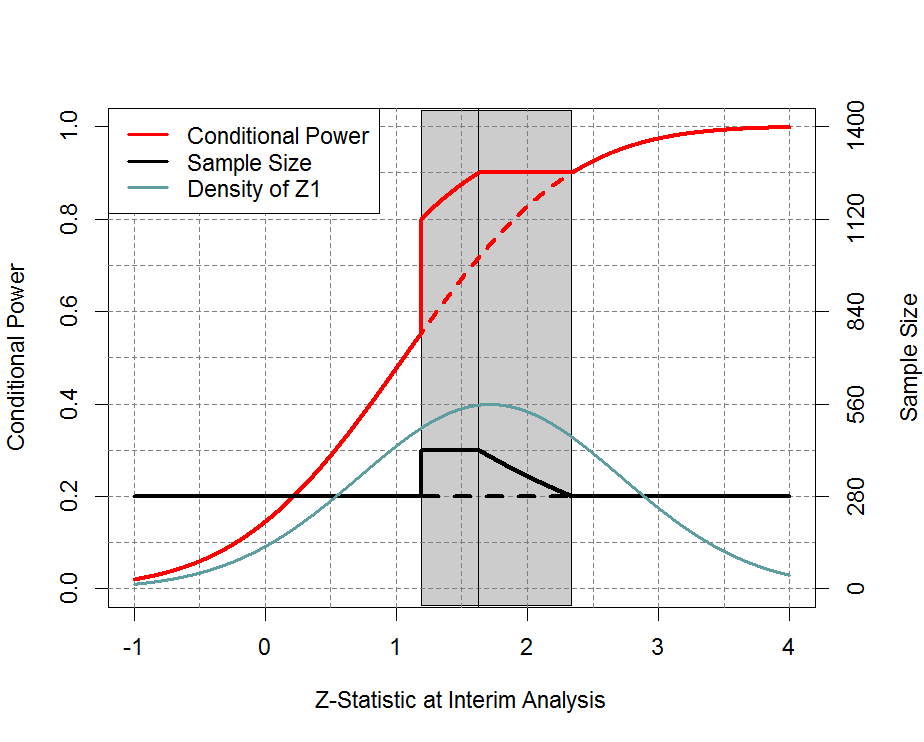

Supplement: Supplementary file 1 — Supporting Information [file BIMJ-61-1175-s001.zip › figures/fig1.tif]

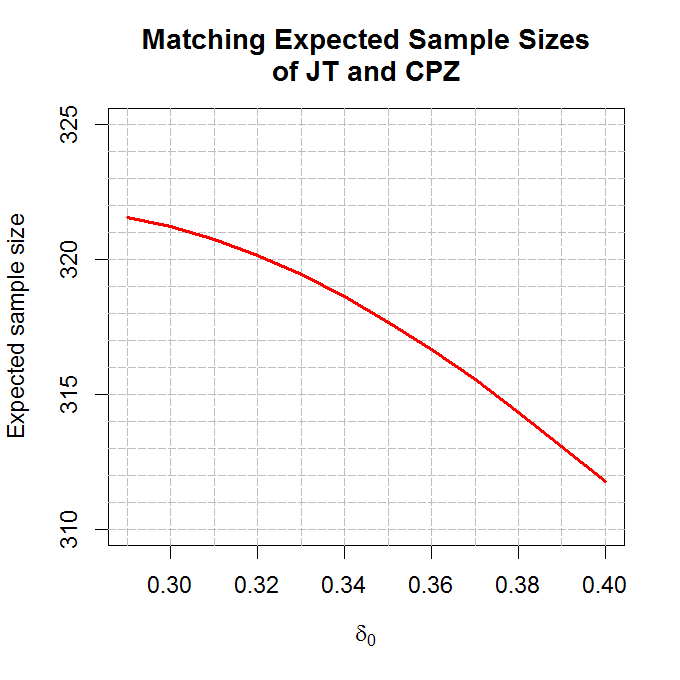

Supplement: Supplementary file 1 — Supporting Information [file BIMJ-61-1175-s001.zip › figures/fig2L.tif]

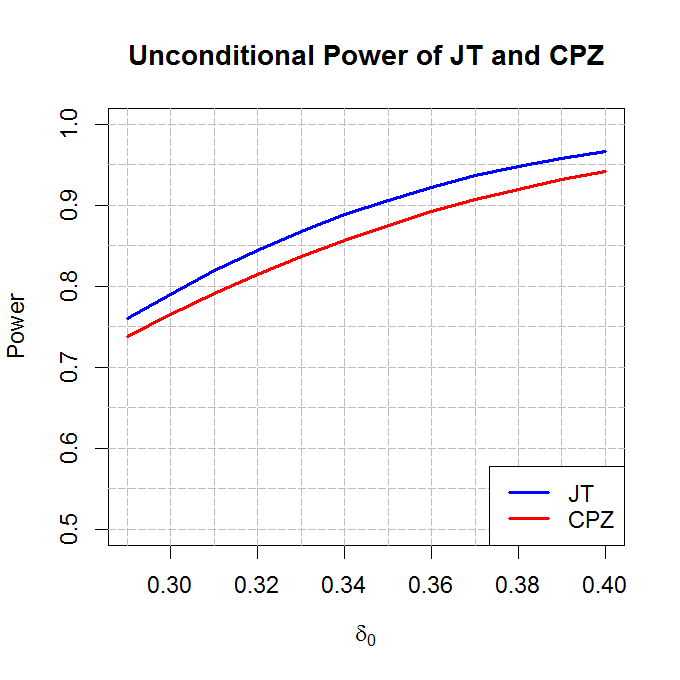

Supplement: Supplementary file 1 — Supporting Information [file BIMJ-61-1175-s001.zip › figures/fig2R.tif]

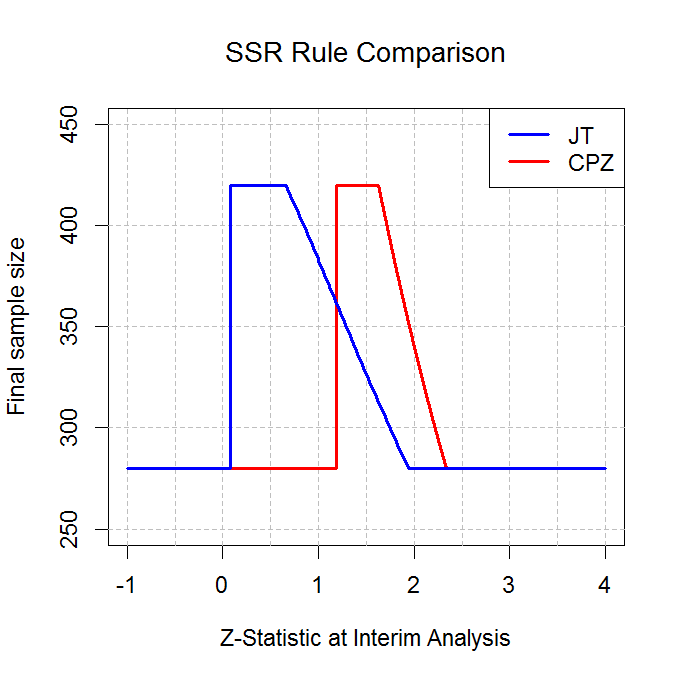

Supplement: Supplementary file 1 — Supporting Information [file BIMJ-61-1175-s001.zip › figures/fig3L.tif]

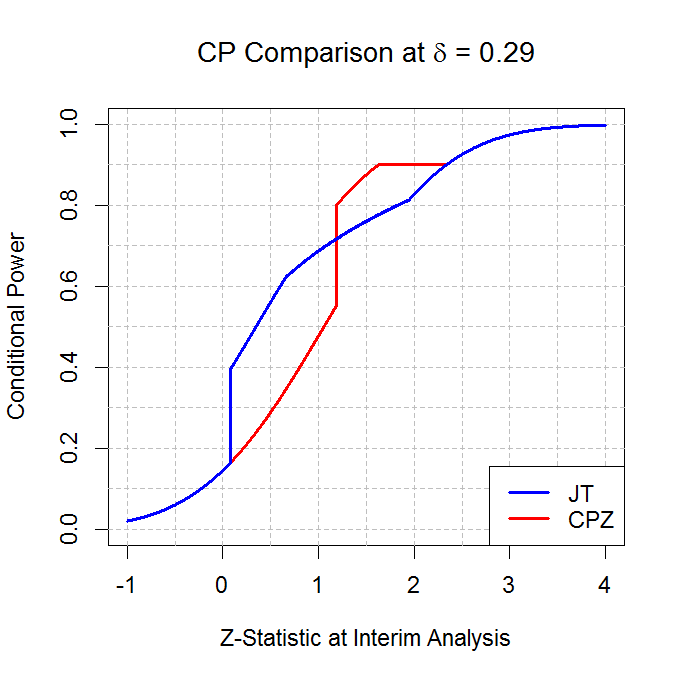

Supplement: Supplementary file 1 — Supporting Information [file BIMJ-61-1175-s001.zip › figures/fig3R.tif]

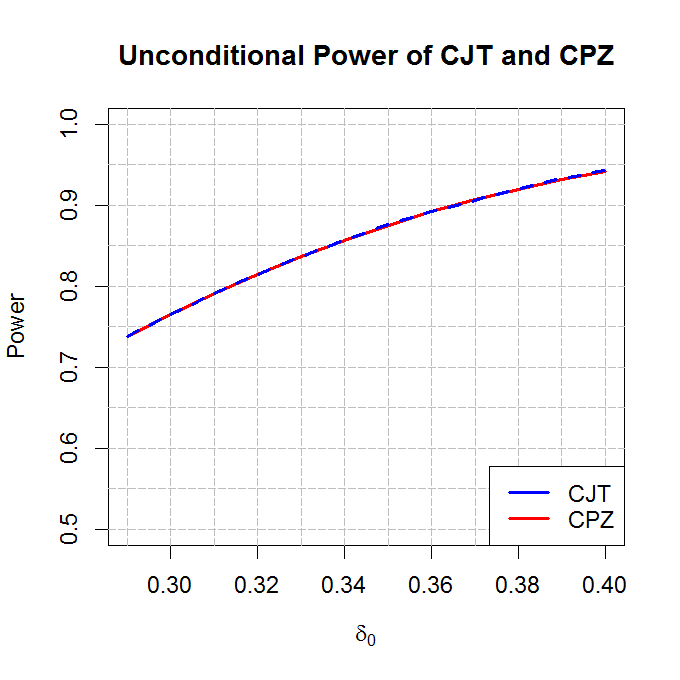

Supplement: Supplementary file 1 — Supporting Information [file BIMJ-61-1175-s001.zip › figures/fig4R.tif]

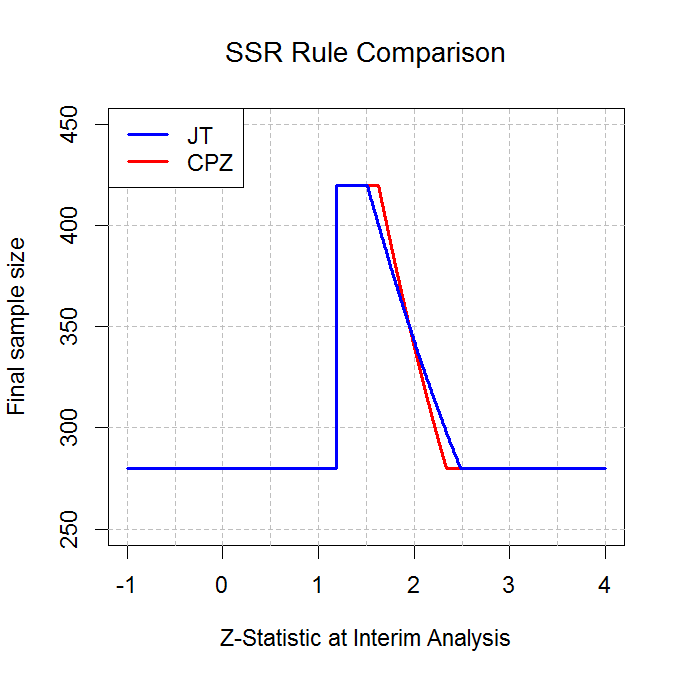

Supplement: Supplementary file 1 — Supporting Information [file BIMJ-61-1175-s001.zip › figures/fig5L.tif]

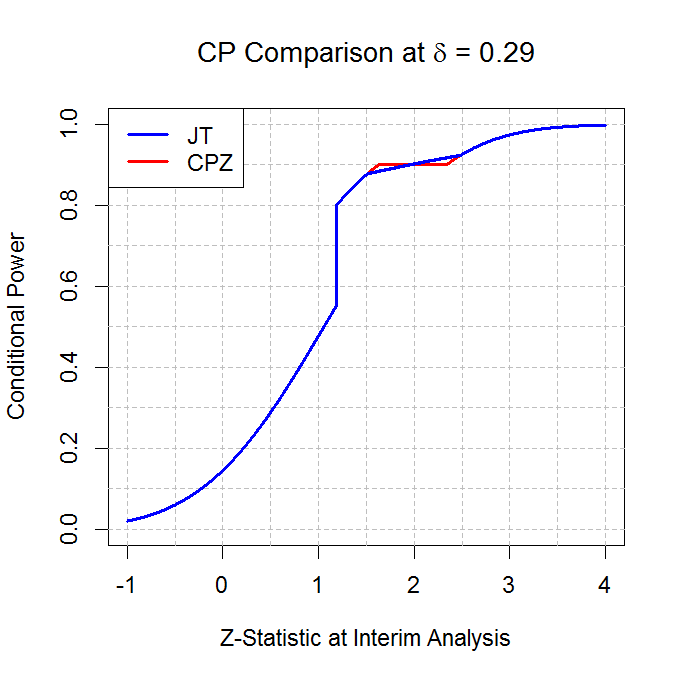

Supplement: Supplementary file 1 — Supporting Information [file BIMJ-61-1175-s001.zip › figures/fig5R.tif]

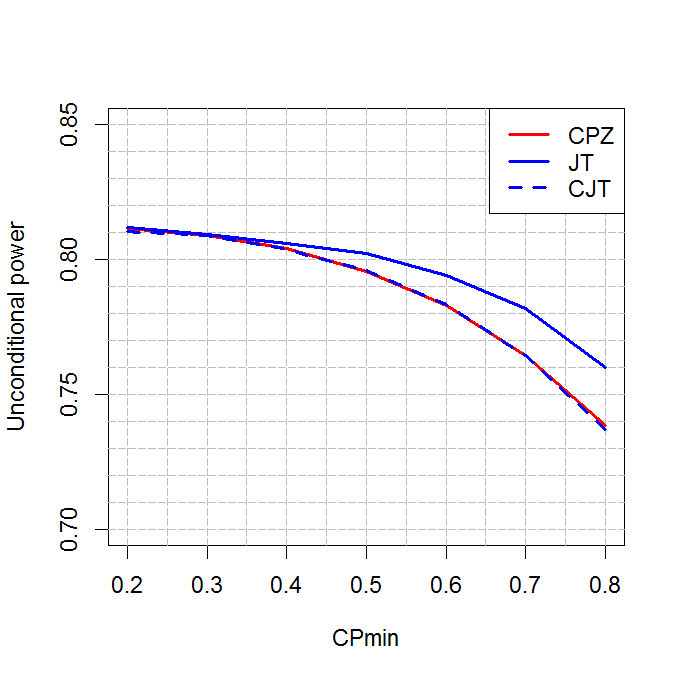

Supplement: Supplementary file 1 — Supporting Information [file BIMJ-61-1175-s001.zip › figures/fig6.tif]
